# The molecular basis of genistein-induced mitotic arrest and exit of self-renewal in embryonal carcinoma and primary cancer cell lines

**DOI:** 10.1186/1755-8794-1-49

**Published:** 2008-10-10

**Authors:** Christian RA Regenbrecht, Marc Jung, Hans Lehrach, James Adjaye

**Affiliations:** 1 Max Planck Institute for Molecular Genetics, Department for Vertebrate Genomics, Ihnestr. 73, D-14195 Berlin, Germany

## Abstract

**Background:**

Genistein is an isoflavonoid present in soybeans that exhibits anti-carcinogenic properties. The issue of genistein as a potential anti-cancer drug has been addressed in some papers, but comprehensive genomic analysis to elucidate the molecular mechanisms underlying the effect elicited by genistein on cancer cells have not been performed on primary cancer cells, but rather on transformed cell lines. In the present study, we treated primary glioblastoma, rhabdomyosarcoma, hepatocellular carcinoma and human embryonic carcinoma cells (NCCIT) with μ-molar concentrations of genistein and assessed mitotic index, cell morphology, global gene expression, and specific cell-cycle regulating genes. We compared the expression profiles of NCCIT cells with that of the cancer cell lines in order to identify common genistein-dependent transcriptional changes and accompanying signaling cascades.

**Methods:**

We treated primary cancer cells and NCCIT cells with 50 μM genistein for 48 h. Thereafter, we compared the mitotic index of treated versus untreated cells and investigated the protein expression of key regulatory self renewal factors as OCT4, SOX2 and NANOG. We then used gene expression arrays (Illumina) for genome-wide expression analysis and validated the results for genes of interest by means of Real-Time PCR. Functional annotations were then performed using the DAVID and KEGG online tools.

**Results:**

We found that cancer cells treated with genistein undergo cell-cycle arrest at different checkpoints. This arrest was associated with a decrease in the mRNA levels of core regulatory genes, *PBK*, *BUB1*, and *CDC20 *as determined by microarray-analysis and verified by Real-Time PCR. In contrast, human NCCIT cells showed over-expression of *GADD45 A *and *G *(growth arrest- and DNA-damage-inducible proteins 45A and G), as well as down-regulation of OCT4, and NANOG protein. Furthermore, genistein induced the expression of apoptotic and anti-migratory proteins p53 and p38 in all cell lines. Genistein also up-regulated steady-state levels of both *CYCLIN A *and *B*.

**Conclusion:**

The results of the present study, together with the results of earlier studies show that genistein targets genes involved in the progression of the M-phase of the cell cycle. In this respect it is of particular interest that this conclusion cannot be drawn from comparison of the individual genes found differentially regulated in the datasets, but by the rather global view of the pathways influenced by genistein treatment.

## Background

Phytoestrogens are a group of plant-derived substances that are structurally and functionally similar to estradiol, therefore mimicking the effects of estrogen [1]. There are 2 major classes of phytoestrogens: the lignans and isoflavones. Isoflavones are the most common form of phytoestrogens and are found in a variety of plants, the greatest dietary source being soy [2-4]. The 2 main isoflavones, genistein and daidzein, are present in soy primarily as β-D-glycosides [1]. Glycosidic bonds are hydrolyzed by glucosidases of the intestinal bacteria in the intestinal wall to produce aglycons [5,6]. The biologically active aglycons [7] are further metabolized to glucuronide conjugates in the intestine and liver.

It is difficult to ascertain the estrogenic activity of phytoestrogens *in vivo *because in addition to the marked inter-individual variability in metabolism and, hence, serum levels obtained, the hormonal milieu of the individual consuming the phytoestrogen likely impacts its effects [8,9]. A systematical review of the literature on the effects of genistein on breast cancer cell growth was performed by de Lemos, and concluded that at low (<10 μmol/L) physiologically relevant levels, genistein stimulates estrogen receptor positive (ER^+^) tumors, while at higher (>10 μmol/L) concentrations, appears to be inhibitory. This has been attributed to the estrogenic properties of genistein being predominant at low levels, while at higher levels, other anticancer actions of phytoestrogens predominate [10]. It is important to note, however, that plasma phytoestrogen levels of over 10 μmol/L are difficult to achieve with dietary intake [7].

The estrogenic activity of phytoestrogens may also depend on their affinity for particular ERs in the body. Phytoestrogens appear to preferentially bind to the ER-β and have sometimes been classified as selective ER modulators (SERMS) [9,11,12]. ER-β may play a protective role in breast cancer development by inhibiting mammary cell growth, as well as inhibiting the stimulatory effects of ER-α [11,13].

Phytoestrogens also have anti-tumor activities that are independent of their estrogenic activity [1,14]. Dietary phytoestrogens have been shown to inhibit proliferation of hormone-independent cell lines [15-17]. For example, genistein has been shown to evoke G2-M cell-cycle arrest in cancer cell lines [18,19] via a multiplicity of interactions, including an inhibition of Cdc2 activity. More recently, genistein has also been linked with the activation of p38 and inactivation of ERK1/2 in human mammary epithelial cells [20,21], indicating that genistein may induce cellular effects via modulations of the mitogen activated protein kinase (MAP kinase)1 signaling cascade.

Pharmacological doses of genistein inhibit the PTK-dependent transcription of c-FOS and subsequent cellular proliferation in estrogen receptor negative (ER^-^) human breast cancer cell lines [22]. Other potential mechanisms that have been reported include phytoestrogen stimulation of the immune system, antioxidant activity, and inhibitory effects on angiogenesis [1,4,14,23-25]. These studies were all carried out *in vitro*.

In this study, we describe the effect genistein has with respect to self-renewal and proliferation of primary cancer cells and embryonal carcinoma cells, which are the stem cells of teratocarcinomas and the malignant counterparts of embryonic stem cells [26,27]. In particular, we show that genistein regulates the expression of a subset of genes and their associated signaling pathways. These results might potentially point into the direction for future cancer stem cell targeting therapies.

## Methods

### Cell culture

NCCIT cells (ATCC, Wesel, Germany, CRL-2073) were cultured in DMEM (GIBCO, Karlsruhe, Germany) with 10% bovine serum (Biochrome, Berlin, Germany), 4 mM L-Glutamin and 1% penicillin-streptomycin. Primary cancer cells (RMS; GBM; HCC-M) were cultured in Quantum 263 Tumor medium (PAA, Pasching, Austria) without antibiotics. Cells were cultured in 5% CO2, 95% air and routinely passaged every 3 days (NCCIT) and 1 week (Cancer cells), respectively.

### Genistein treatment

Genistein (Roth, Karlsruhe, Germany) was prepared as a concentration of 50 mg/ml in DMSO. Cells were counted using Trypan-blue (Sigma, Munich, Germany) and 3 × 105 cells were seeded in a 24 well plate and cultured for 24 h enabling attachment to the surface, and then treated with 50 μM genistein for a further 48 h. Incubation with corresponding amount DMSO served as control.

### Assessment of morphological changes

Cell morphology was investigated using an inverted phase contrast microscope (Zeiss LSM 510 Meta; Carl Zeiss, Jena) and a CCD Camera.

### Assessment of Mitosis

Immunofluorescence staining with antibody (1:250) to phosphorylated histone-3 (H3P, Upstate Biotechnology, NY), a mitosis-specific marker was performed on treated and untreated cells. Cells were identified with antibody to alpha-Tubulin (DM1A, ABCAM, Cambridge, UK) as above. Anti-rabbit rhodamine (Molecular Probes, OR) was used as the secondary antibody for the H3P antibody, and anti-mouse IgM FITC (Sigma) was used as the secondary against DM1A. Nuclei were stained using DAPI. The number of nuclei staining positive for H3P were counted per field at × 40 magnification under fluorescent field optics. The total number of nuclei per field was counted, and a mitotic index was computed as the ratio of H3P-positive nuclei to total nuclei. The mitotic indices for at least 2000 cells were averaged.

### Real-time PCR

RNA was reverse transcribed using MMLV (USB, OH) and oligo-dT priming. Real-time RT-PCR was carried out on an Applied Biosystems 7900 PCR machine in 20 μl reactions consisting of 10 μl of SYBR Green PCR mix (ABI, CA), 0.375 μM of each primer, and diluted cDNA. All primer pairs used were confirmed to approximately double the amount of product within one cycle and to yield a single product of the predicted size. For primer sequences see Additional file 1. Relative mRNA levels were calculated using the comparative Ct method (ABI instruction manual) and presented as a % of biological controls. ACTB and GAPDH transcript levels were confirmed to correlate well with total RNA amounts and therefore used for normalisation.

### Western-blot analysis

Western-blotting was performed according to standard procedures and using chemiluminescence detection (ECL – Amersham, Buckinghamshire, UK). Antibodies used were Santa Cruz (Heidelberg, Germany) sc-8629 (OCT4), R&D AF1997 (NANOG), Santa Cruz sc-17320 X (SOX2), Ambion (Darmstadt, Germany) #4300 (GAPDH), Calbiochem (Darmstadt, Germany) #401504 (HRP-linked), as well as Amersham NA9340 and NA9310 (HRP-linked).

### Chip hybridisations and analysis of whole-genome expression data

Biotin-labelled cRNA was generated employing a linear amplification kit (Ambion #IL1791) using 300 ng of DNA-free, quality-checked total RNA as template. Chip hybridisations, washing, Cy3-streptavidin (Amersham Biosciences) staining, and scanning was performed on the Illumina (CA, USA) BeadStation 500 platform employing reagents and following protocols supplied by the manufacturer. cRNA samples were hybridised as biological duplicates on Illumina human-8 BeadChips. Samples to be hybridised were harvested 2 days after induction with 50 μM genistein. All basic expression data analyses were carried out using the manufacturer's BeadStudio 3.0 software. Raw data were background-subtracted and normalised using the "rank invariant" algorithm. Values below the detection limit were arbitrarily set to the level of threshold detection in order to avoid nonsense values for expression ratios. Significantly differentially expressed genes had to have a fold change of at least 50% with a p-value < 0.01. Pathway and Gene Ontology analyses were carried out using DAVID 2006 [28]. In both cases, we used GenBank accession numbers represented by the corresponding chip oligonucleotides as input.

### Comparison with datasets from selected publications

To compare our data with that of previous studies, we extracted all genes detected as differentially expressed in the respective studies and deleted duplicate genes names from the lists. Pathway and Gene Ontology analyses were carried out as described above.

## Results

### Sensitivity of cell lines to μM concentrations of genistein

**Figure 1:**
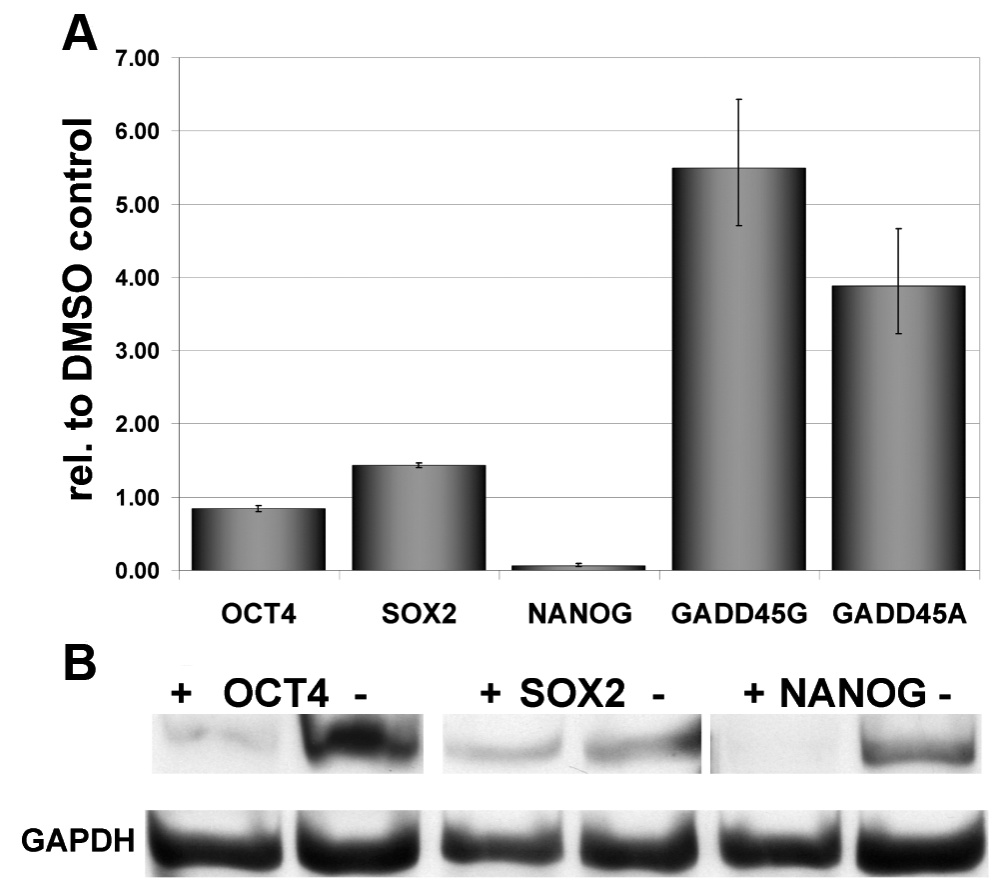
**Expression of key pluripotency associated genes after induction with genistein**. (A) Real-Time PCR showing upregulated expression of GADD45A and G, and a drastic down-regulation of NANOG. (B) Western-blot showing down regulation of NANOG. (-) non-treated DMSO control, (+) genistein treated NCCIT cells.

Human embryonic carcinoma (NCCIT) cells were treated with 50 μM, 100 μM genistein and DMSO as control. Further growth was carried out for 48 h, RNA isolated and the expression of *GADD45A *and *GADD45G *analysed by Real-Time PCR (Figure 1). As shown in Figure 1A, genistein induces transcription of these genes as well as down regulation of *NANOG*. To test, if genistein treatment also alters the protein-levels of known markers of pluripotency, we performed Western-blot analysis of OCT4, SOX2 and NANOG in treated and untreated NCCIT cells. As shown in Figure 1B, decreased protein levels of OCT4 and NANOG correlate with the results from RT-PCR analysis (Figure 1A).

### Genistein treatment induces mitotic arrest in NCCIT and cancer cells

To investigate the effect of genistein treatment on signaling pathways operative in different solid human tumors, we used the following human cell lines: HS68 (fibroblasts), NCCIT (embryonal cancer cell line), U373 (glioma cell line), MCF7 (breast cancer cell line), HCC (hepatocellular carcinoma), HCC-M (metastasis of HCC) GBM1207 (primary glioblastoma), and eRMS (embryonic Rhabdomyosarcoma). All cell lines were treated with 50 μM genistein and analysed by phase contrast microscopy after 48 h.

**Figure 2:**
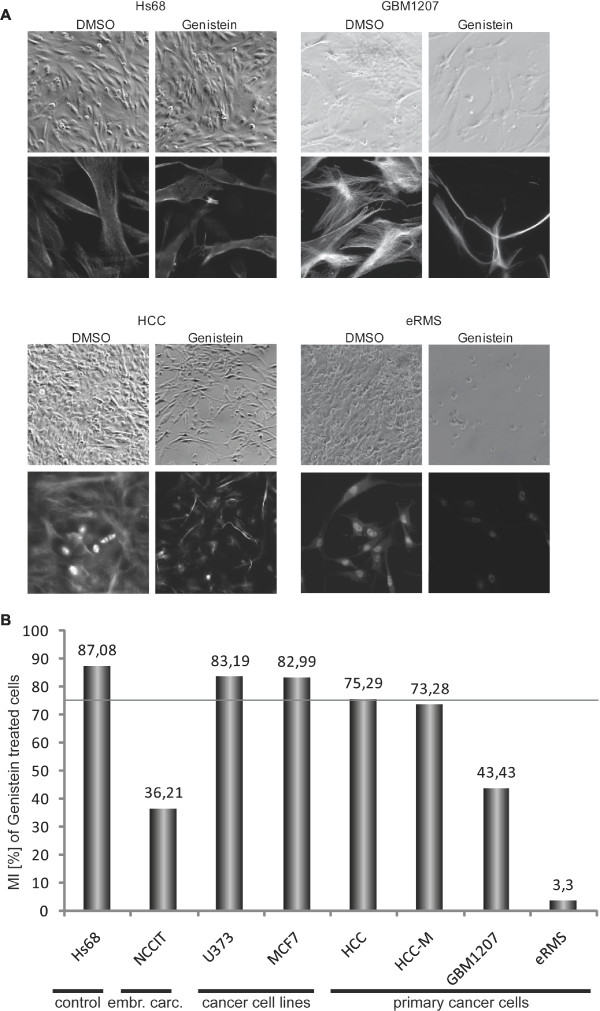
**Morphology and mitotic index of genistein treated cells**. (A) Phase-contrast and immunofluorescence micrographs of untreated (DMSO) and treated (50 μM genistein/48 h) cells. Hs68 cells serving as negative control did not show changes in morphology upon treatment. Genistein-treated cancer cell lines in comparison (GBM1207, HCC, HCC-M) show clear morphological changes, resembling a more fibroblast-like type. (B) The effect of genistein treatment on 8 different cell lines was investigated by calculating the mitotic index of each sample. Frequency of mitosis in each matching DMSO-control was set as 100% for each cell line and the relative decrease upon treatment was calculated. The threshold for significance was set to 75% mitoses (grey line). Hs68 cells served as negative control (MI >87% after treatment). Interestingly, both cell lines (MCF7 and U373) also showed only mild response to the treatment and mitotic indices were preserved at levels >82% compared to the corresponding control. In NCCIT cells as well as primary cancer cells, the observed effect on levels of mitosis was significantly high. Mitosis rates were as low as 3,3% (eRMS); 36,21 (NCCIT); 43,43% (prim. GBM); 73,28 (metastasis of HCC).

We frequently observed a decrease in cell density after 48 h of genistein treatment in all primary cell lines analysed (Figure 2A). To investigate if this genistein-induced reduction is caused by mitotic arrest, we performed immunofluorescence staining for phosphorylated histone H3 (H3P), which is a well characterized mitotic protein. Reduction in mitotic index was found predominantly in the primary cell lines (Figure 2B). Foreskin cells and transformed cell lines had a low reduction of the mitotic index, NCCIT cells and primary cell lines had moderate to severe reductions in MI levels (Figure 2B). Interestingly, poorly differentiated, high-grade cancer such as the glioblastoma cell line showed the mildest reduction. The data presented is that of independent duplicates showing the same pattern of reduction.

In addition to reduced cell numbers, we observed a dramatic change in morphology in all cancer cell lines. We identified an increasing number of individual cells exhibiting cytoplasmic condensation and nuclear polymorphism (pleiomorphy). The embryonic fibroblast foreskin cell line Hs68, did not show visible morphological changes (Figure 2A).

### Global gene expression analysis

RNA isolated from the cell samples was used for global gene expression analysis employing the Illumina platform and following the manufacturer's recommendation. The reproducibility between replicate samples was assessed by calculating correlation coefficients. The values ranging between 0.98 and 0.99 for biological replicates indicate a high degree of reproducibility (see Additional file 2).

**Figure 3:**
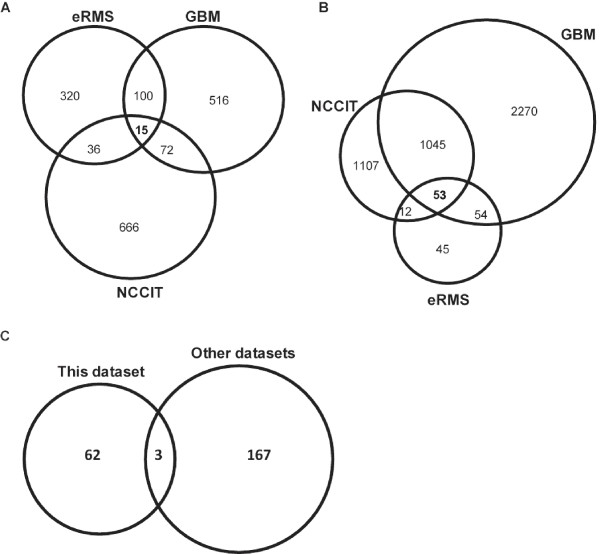
**Cell-type comparison of differentially regulated genes after Genistein treatment**. Venn diagram illustrating distribution of down-regulated (A) and up-regulated (B) genes after genistein treatment compared to their respective DMSO-controls. The description of the common regulated genes is given in Additional file 3. (C) Venn diagram of differentially regulated genes identified in our study, compared to [29-31].

We investigated the primary cancer cell lines GMB1207, eRMS and the NCCIT cells for differential expression of genes upon genistein treatment. Based on a detection score = 0.99 and a p-value < 0.01 in all cell lines (genistein vs. DMSO-control), we observed that in GBM1207 cells, 3419 genes were up-regulated (>1.5-fold) and 516 genes down-regulated (<0.66-fold). Compared to NCCIT and eRMS, GBM1207 presented the largest set of differentially regulated genes. In eRMS cells, we found 161 genes up- and 471 genes down-regulated, compared to the NCCIT cells, where 2214 genes are significantly up-regulated, with 789 genes showing down-regulated expression. The overlap of differentially expressed genes between treated and untreated cells is shown in Figure 3. We have compared the common down-regulated genes between the cell lines (3A), the up-regulated genes (3B) and compared all differential regulated genes to that of previously published studies in long-term cell lines (3C).

### Analysis of genistein-dependent pathways

To identify common signaling and metabolic pathways in cancer cells that are modulated by genistein, we merged the datasets of all the primary cancer cell lines and compared the differentially expressed genes. We found 15 genes that were down-regulated and 53 genes with increased expression (see Additional file 3). David Pathway annotation analysis was then carried out with these 53 genes as input. The top five most enriched KEGG pathways are, Cell-cycle (hsa04110), p53 signaling pathway (hsa04115), MAPK signaling pathway (hsa04010), regulation of actin cytoskeleton (hsa04810), DNA polymerase (hsa03030). Up-regulated genes included members of the TNF-superfamily (e.g. *TNFSF9*), p53-signaling cascade (e.g. *DDIT3*), and apoptosis (e.g. *PDCD6IP*). Amongst the differentially regulated genes were predominantly those involved in regulating the progression of the cell-cycle. GO-analysis identified the cellular localization and biological function of the identified proteins; these are nucleus (32%), microtubule cytoskeleton (20%), the spindle (10%), and the presence of condensed chromosomes (4%). GO annotation also revealed significantly regulated genes which identified factors responsible for driving differentiation of NCCIT and cancer cells and factors responsible for maintaining the undifferentiated state. These factors are involved in the following biological processes mitotic cell-cycle, cell proliferation, and cell-cycle checkpoint, regulating the cell-cycle process, DNA-metabolism, response to DNA damage stimulus, and DNA repair. These fulfill important functions for maintaining chromosomal integrity.

The largest group of genes encoding proteins that regulate cell proliferation (ie mitotic index) came from within the genistein treated primary cancer cells.

**Figure 4:**
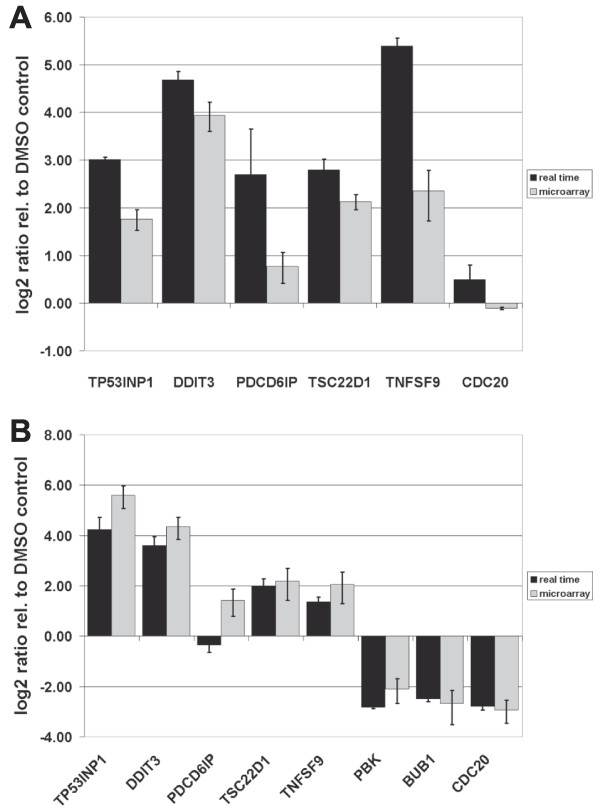
**Real-Time PCR validation of selected target genes**. (A) NCCIT cells (B) GBM1207 cells. In the NCCIT cell line, the expression of the mitotic M-phase related gene, PBK, was not detected and BUB1 was not significantly regulated based on the Illumina microarray analysis.

The reduction of mitotic indices was confirmed by the RNA-expression data. In order to validate the array generated data, we performed Real-Time PCR analysis of selected genes using NCCIT and GBM1207 cells. These genes included some of the master-regulators of the cell-cycle like *CDC20*, *BUB1*, and *PBK*. As expected, we observed a reduction of expression for these genes as compared to the control (Figure 4A, B).

As shown in Table 1, only 5 genes are involved in the I-phase and G2-phase transition, whilst 20 genes are involved in M-phase regulation. Amongst these are regulators of mitosis e.g. *BUB1*, *CDC20*, and *PBK*, which are known to play important roles in the ontology of various types of cancers. Unlike the NCCIT cells, expression of *OCT4*, *SOX2 *and *NANOG *could not be detected in any primary cancer cell line tested. Real time validation with GBM1207 confirmed this result.

**Table 1:** Cell cycle related genes differentially regulated upon genistein treatment

**Symbol**	**Definition**
**Interphase of the mitotic cell cycle**	

CCNB1	cyclin B1
CDC2	cell division cycle 2, G1 to S and G2 to M, transcript variant 1
CDCA5	cell division cycle associated 5
	
**G2-phase of the mitotic cell cycle**	

CENPF	centromere protein F, 350/400 ka (mitosin)
GTSE1	G-2 and S-phase expressed 1
	
**M-phase of the mitotic cell cycle**	

ASPM	asp (abnormal spindle)-like, microcephaly associated (Drosophila)
BUB1	BUB1 budding uninhibited by benzimidazoles 1 homolog (yeast)
BUB1B	BUB1 budding uninhibited by benzimidazoles 1 homolog beta (yeast)
CCNA2	cyclin A2
CCNB2	cyclin B2
CDC20	CDC20 cell division cycle 20 homolog (S. cerevisiae)
CENPE	centromere protein E, 312 kDa
CIT	citron (rho-interacting, serine/threonine kinase 21)
DLG7	discs, large homolog 7 (Drosophila)
HCAP-G	chromosome condensation protein G
KIF2C	kinesin family member 2C
PBK	PDZ binding kinase
SPAG5	sperm associated antigen 5
TTK	TTK protein kinase
UBE2C	ubiquitin-conjugating enzyme E2C, transcript variant 6
UBE2C	ubiquitin-conjugating enzyme E2C, transcript variant 5
UBE2C	ubiquitin-conjugating enzyme E2C, transcript variant 2

Comparison with previously published data

**Figure 5:**
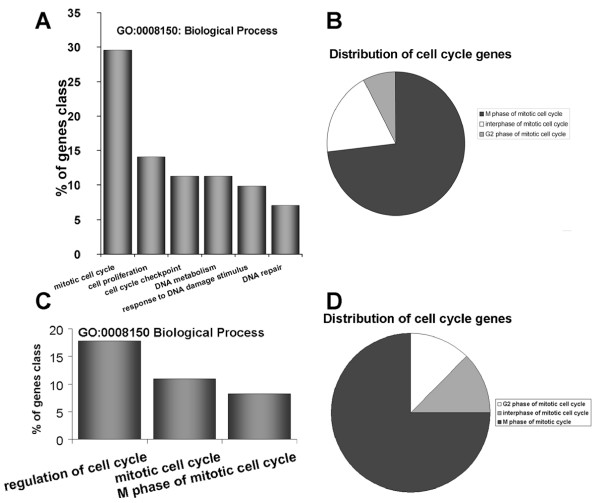
**Distribution of genistein induced cell-cycle regulating genes**. Amongst the down-regulated targets (> 0.66), genes of the M-phase of the mitotic cycle are significantly over-represented. (A) GO clustering for biological processes (B) Pie chart representing the relative number of M-phase related genes. (C) GO clustering for biological processes from the datasets of [29-31]. (D) Pie chart illustrating the relative numbers of genistein targeted M-phase genes. The corresponding table shows examples of genes specified in each GO cluster

We compared our data with that of previously published datasets related to genistein [29-31] dependent expression patterns. Because the different pre-requisites used to carry out these studies, we included all genes significantly differentially expressed, regardless if they were over- or under-expressed. This analysis revealed a common set of only three genes differentially expressed between the datasets (Figure 3C). *DCXR*, *NQO1*and *SCD *are involved in key metabolistic pathways, thus suggesting their important role in genistein-processing and translation of the stimulus into a cellular response. Another important finding of the comparison between these gene-sets is that on a pathway level all gene-sets point towards the mitotic cell cycle (Figure 5), specifically towards the M-phase regulating genes.

## Discussion

Cancer is a complex disease, characterized by deregulated proliferation, and aberrant cell-cycle control. This is an important difference between normal and malignant cells [32-34].

Previous experimental work addressing the effects of genistein on cell proliferation and differentiation were performed using prolonged-cultured, transformed cell lines. These earlier findings, though informative, have short comings with respect to the genomic integrity of the cells used for these analyses. We have shown that genistein applied to low passage cultured cells has a noticeable effect on the transcription of common key regulators of cell-cycle progression. In terms of the mechanism(s) of action of genistein, NF-kB-mediated repression of *GADD45A *and *G *expression has been shown to be essential for cancer cell survival [35]. Furthermore, *GADD45A *expression has been shown to be induced by genistein treatment of human prostate cancer cell lines [36]. To test if genistein also imparts similar effects in other cancer cells, we initially used the embyonal carcinoma cell line (NCCIT) which has properties of cancer cells as well as pluripotent cells [26,27]. GADD45G and GADD45A are regulators of the cell-cycle at the G2/M transition [37] and act as tumor suppressors [38]. The direct effect of genistein on GADD45 gene expression has been shown before [36]. In this study, we have verified this effect for *GADD45G *and *GADD45A*. Furthermore, *GADD45G *has been shown to be a negatively regulated, direct downstream target of OCT4 [27,39,40]. Indeed, genistein treatment of NCCIT cells led to the induction of *GADD45A *and *GADD45G *expression, as shown previously with other cancer types. Additionally, we noticed a reduction in *NANOG *transcription but not that of *OCT4 *and *SOX2*. A reduced level of *NANOG *could not be linked to a differentiation phenotype, but rather to reduced proliferation in NCCIT cells [27]. As shown before, down-regulation of OCT4 leads to the down-regulation of NANOG, we assume that our observed decrease in the transcript level of *NANOG *is a downstream effect of genistein-induced depletion of OCT4 protein [41]. Furthermore, a decrease in OCT4 and NANOG protein was detected. We speculate that genistein treatment might indirectly down-regulate OCT4 expression, possibly mediated by the up-regulated expression of GADD45G. Our investigation was designed to evaluate the effects of genistein on cellular proliferation and changes in cell morphology in primary cancer cells derived from tumour tissue and cultured for only a brief period. Employing both a cell culture system and global expression analysis, we elucidated effects of genistein which are shared between 3 different primary cancer cells and the embryonal carcinoma cell line -NCCIT.

Evidence for genistein-induced effects was the obvious reduction of cells in the treated sample, and closer examination showed a complete re-structuring of the morphology of the cells towards that of fibroblasts. This observation is unclear at the moment because we could not see an increase in the expression of the epithelial markers, *EPCAM*, *CDH1 *or *KRT10 *in NCCIT cells, unlike the primary cancer cell lines.

As anticipated, the Gene Ontology (GO) and the KEGG pathway analysis revealed an over-represented number of genes involved in check-point control of the cell-cycle and associated signaling pathways (p53- and ubiquitin-proteasome-pathway). The most over-represented pathway in our studies was the cell-cycle, specifically the control of cell-cycle progression (Figure 4).

The cell-cycle regulates cell growth and division to ensure that every cell receives a complete set of chromosomes. Mis-segregation of chromosomes may lead to genomic instability, which can be found in a wide variety of tumors, such as colon, breast, prostate, oropharynx or lung cancer, leukemia and lymphoma [42-49].

The expression pattern of CYCLINS dictates the point in the cell-cycle at which they act. *CYCLIN A *and *B*, which were down-regulated in NCCIT, glioblastoma and rhabdomysarcoma cells are associated with both CDK1 and CDK2, which govern the transition through G1-phase of the cell-cycle, past the restriction point.

Down-regulation of CYCLIN A could be a potential target for cancer treatment, because its over-expression is known to feedback onto p53 and is associated with an increased risk of cancer in humans [50].

The G1/S checkpoint appears to be the most crucial step in the genesis and progression of cancer [51,52]. It is triggered by the kinase, CHK1, which we found down-regulated in NCCIT cells and in the GBM and the eRMS cell line. This could be a possible explanation for the decrease in mitosis seen upon genistein treatment.

A specific mechanism which guarantees genomic integrity is the control of the spindle assembly checkpoint [53,54]. This is under the control of BUB1, believed to function primarily on the mitotic spindle checkpoint. The ultimate target of the checkpoint is inhibition of the anaphase promoting complex (APC), which is essential for cell differentiation or accurate DNA replication in the following S phase [55,56]. The affinity of activators of the APC is regulated by CDC20; although it is controversial whether phosphorylation of CDC20 is necessary for APC activation in human cells [57-59], it is required for its inhibition by the spindle checkpoint [60].

The PDZ binding kinase (PBK), which is up-regulated in various neoplasms [61,62] and in genistein-treated cells, has been the focus of attention, especially the elucidation of its role in malignant conversion and as a possible therapeutic target in numerous types of cancers. Although PBK expression has been shown to correlate with proliferation of cancer cells [63], PBK silencing does not prevent progression through the cell-cycle. However, cells with decreased PBK expression have impaired p38 activation after growth-factor stimulation. This correlates with decreased motility, and after treatment with DNA-damaging agents, results in increased DNA damage and sensitivity towards genotoxic agents [63]. These cells also displayed reduced long-term proliferation and a reduction in anchorage-independent growth.

PBK expression has been shown to be down-regulated during induced growth arrest in G2/M phase and to be regulated by cell-cycle-specific transcription factors such as E2F and CREB/ATF [64]. Aberrant entry into the mitotic phase has been shown to be due to down-regulation of p53 caused by direct physical interaction with PBK [65].

Inactivation of the pRb and p53 pathways at the G1/S transition is a fundamental requirement for the genesis of most human cancers. This finding further provides the link to p53-signaling and the ubiquitin-proteasome signaling, both categories found over-represented in the KEGG analysis of common genes between genistein treated cell lines.

As shown in Figure 3, the overlap of the genes found in our dataset compared to that of others [29-31] recovers only three genes. These three genes were *DCXR, NQO1 SCD*, which are all involved in metabolism. *DCXR *and *NQO1 *have been implicated in various tumors, thus not specifically linked to genistein treatment. On the other hand, Stearoyl-CoA desaturase (SCD) seems to be of particular interest in investigating the effects of genistein. SCD is an iron-containing enzyme that catalyzes a rate-limiting step in the synthesis of unsaturated fatty acids and has been implicated in the regulation of cell growth and differentiation through effects on cell membrane fluidity and signal transduction [66,67].

A comparison of the Gene Ontology of the other datasets to ours revealed an astonishing similarity between the studies. For example, the percentage-distribution of genes accompanying the various phases of the cell cycle is more or less identical, with about 75% of genes involved in M-phase transition.

In summary, it appears that genistein has multiple effects. Depending on the cell line and the phase of the cell cycle at the time of treatment, cells that may have already passed G1/S checkpoint or the intra-S-checkpoints were arrested at the G2/M checkpoint by differential reduction of CDC2 expression as shown in NCCIT cells and primary GBM. The G2/M checkpoint prevents cells from entering mitosis when they experience DNA damage during G2 or when they carry unrepaired DNA from G1 or S to progress into G2 of the cell cycle [68]. The critical targets of p53 at G2/M are p21, GADD45A and GADD45G, which induce the dissociation of the CDC2 and CYCLIN complex [68,69]. In addition, p53 appears to repress the transcription of *CDC2 *and *CYCLIN B*. Two isoforms of MAP kinase, p38 alpha and gamma, have also been implicated in the G2/M checkpoint [70].

## Conclusion

On a broad basis, our results from low-passage primary cancer cells may explain the observations made by others using long-term cultured cells. But more importantly, this study provides insights into the molecular mechanisms underlying the morphological changes elicited by genistein treatment of embryonal carcinoma and distinct primary and transformed cancer cell lines.

From the comparisons of distinct datasets obtained under various conditions in terms of concentration and induction-time of genistein, as well as varying cell culture conditions, it seems that the molecular mechanisms triggered by the treatment are very robust and universal. Collectively, our findings provide clear evidence that genistein has a specific effect on major cell-cycle regulatory genes and their associated pathways, which include apoptosis (down-regulation of p53) and motility (by cross-signaling to p38; MAPK).

In conclusion, genistein may be a potent cell-cycle regulating drug targeting the M-phase, both in cell lines and primary patient-derived cancer cells from various tumor entities. But still, enthusiasm has to be dampened, because these doses will not be attained pharmacologically. However, if this pitfall of high dose levels can be overcome – for example by adjuvant administration of other compounds making cancer cells more sensitive towards genistein treatment, genistein may well justify emerging phase I and II trials of this potent cell-cycle regulating compound in the treatment of cancer patients.

## Competing interests

The authors declare that they have no competing interests.

## Authors' contributions

CRAR performed the cell biological studies, data analysis and drafted the manuscript. MJ performed the molecular studies, data analysis and drafted the manuscript. HL is the Head of the Department of Vertebrate Genomics at the Max Planck Institute for Molecular Genetics. JA was responsible for the co-ordination and supervision of the entire study.

## Pre-publication history

The pre-publication history for this paper can be accessed here:



## Supplementary Material

Additional File 1 **Sequences of primers used for Real-Time PCR.** Sequences of primers used for Real-Time PCR validation of selected genes. Click here for file

Additional File 2 **Summary of the expression data and the corresponding gene annotations.** Expression profiles of genistein treated cell lines and their respective controls (GBM1207, eRMS, NCCIT). Click here for file

Additional File 3 **Common set of differentially regulated genes.** 53 genes were up-regulated and 15 genes were commonly down-regulated in the investigated NCCIT, eRMS and GBM1207 cell lines. Click here for file
